# Postoperative Innate Immune Dysregulation, Proteomic, and Monocyte Epigenomic Changes After Colorectal Surgery: A Substudy of a Randomized Controlled Trial

**DOI:** 10.1213/ANE.0000000000007297

**Published:** 2024-10-25

**Authors:** Kim I. Albers-Warlé, Leonie S. Helder, Laszlo A. Groh, Fatih Polat, Ivo F. Panhuizen, Marc M. J. Snoeck, Matthijs Kox, Lucas van Eijk, Leo A. B. Joosten, Mihai G. Netea, Yutaka Negishi, Musa Mhlanga, Christiaan Keijzer, Gert-Jan Scheffer, Michiel C. Warlé

**Affiliations:** From the * Department of Anesthesiology, Radboudumc, Nijmegen, the Netherlands; † Department of Surgery, Radboudumc, Nijmegen, the Netherlands; ‡ Department of Surgery, Canisius Wilhelmina Hospital, Nijmegen, the Netherlands; § Department of Anesthesiology, Canisius Wilhelmina Hospital, Nijmegen, the Netherlands; ‖ Department of Intensive Care Medicine, Radboudumc, Nijmegen, the Netherlands; ¶ Department of Internal Medicine, Radboudumc, Nijmegen, the Netherlands; # Department of Immunology and Metabolism, Life and Medical Sciences Institute, University of Bonn, Bonn, Germany; ** Department of Medical Genetics, Iuliu Hatieganu University of Medicine and Pharmacy, Cluj-Napoca, Romania; †† Department of Biology, Radboudumc, Nijmegen, the Netherlands.

## Abstract

**BACKGROUND:**

Colorectal surgery is associated with moderate-to-severe postoperative complications in over 25% of patients, predominantly infections. Monocyte epigenetic alterations leading to immune tolerance could explain postoperative increased susceptibility to infections. This research explores whether changes in monocyte DNA accessibility contribute to postoperative innate immune dysregulation.

**METHODS:**

Damage-associated molecular patterns (DAMPs) and ex vivo cytokine production capacity were measured in a randomized controlled trial (n = 100) in colorectal surgery patients, with additional exploratory subgroup proteomic (proximity extension assay; Olink) and epigenomic analyses (Assay for Transposase-Accessible Chromatin [ATAC sequencing]). Monocytes of healthy volunteers were used to study the effect of high-mobility group box 1 (HMGB1) and heat shock protein 70 (HSP70) on cytokine production capacity in vitro.

**RESULTS:**

Plasma DAMPs were increased after surgery. HMGB1 showed a mean 235% increase from before- (preop) to the end of surgery (95% confidence interval [CI] [166 – 305], *P <* .0001) and 90% increase (95% CI [63–118], *P =* .0004) preop to postoperative day 1 (POD1). HSP70 increased by a mean 12% from preop to the end of surgery (95% CI [3–21], not significant) and 30% to POD1 (95% CI [18–41], *P <* .0001). Nuclear deoxyribonucleic acid (nDNA) increases by 66% (95% CI [40–92], *P <* .0001) at the end of surgery and 94% on POD1 (95% CI [60–127], *P <* .0001). Mitochondrial DNA (mtDNA) increases by 370% at the end of surgery (95% CI [225–515], *P <* .0001) and by 503% on POD1 (95% CI [332–673], *P <* .0001). In vitro incubation of monocytes with HSP70 decreased cytokine production capacity of tumor necrosis factor (TNF) by 46% (95% CI [29–64], *P <* .0001), IL-6 by 22% (95% CI [12–32], *P =* .0004) and IL-10 by 19% (95% CI [12–26], *P =* .0015). In vitro incubation with HMGB1 decreased cytokine production capacity of TNF by 34% (95% CI [3–65], *P =* .0003), interleukin 1β (IL-1β) by 24% (95% CI [16–32], *P <* .0001), and IL-10 by 40% (95% CI [21–58], *P =* .0009). Analysis of the inflammatory proteome alongside epigenetic shifts in monocytes indicated significant changes in gene accessibility, particularly in inflammatory markers such as CXCL8 (IL-8), IL-6, and interferon-gamma (IFN-γ). A significant enrichment of interferon regulatory factors (IRFs) was found in loci exhibiting decreased accessibility, whereas enrichment of activating protein-1 (AP-1) family motifs was found in loci with increased accessibility.

**CONCLUSIONS:**

These findings illuminate the complex epigenetic modulation influencing monocytes’ response to surgical stress, shedding light on potential biomarkers for immune dysregulation. Our results advocate for further research into the role of anesthesia in these molecular pathways and the development of personalized interventions to mitigate immune dysfunction after surgery.

**KEY POINTS**

**Question:** Does colorectal surgery induce epigenetic changes in monocytes that lead to postoperative immune tolerance?

**Findings:** Postsurgery, patients exhibited elevated plasma damage-associated molecular patterns (DAMPs) and monocytes showed altered cytokine production and significant epigenetic changes in genes related to inflammation.

**Meaning:** The study highlights the role of epigenetic regulation of monocytes in response to surgical stress, suggesting new potential biomarkers for immune dysregulation and paving the way for personalized treatment to reduce postoperative complications.

Colorectal surgery is associated with a high postoperative complication rate, 27% of patients develop moderate to severe 30-day complications.^1^ The majority of these complications are infectious, not only surgical-site and wound infections, but also distant infections such as pneumonia and urinary tract infections.^1,2^ The capacity to elicit an inflammatory response is an important predictor of postoperative complications and survival after colorectal surgery.^3^ It is well known that the early postoperative immune response is predominantly suppressive, leaving patients more vulnerable to secondary infections.^4^ Moreover, immune dysregulation plays an important role in growth and spread of many cancers, including colorectal carcinoma. Early postoperative innate immune dysregulation and the immune phenotype associated with a higher risk of infections are currently not well understood.

Our research group recently published that decreasing surgical tissue injury by lowering intraabdominal pressure (IAP) during laparoscopic colorectal surgery can preserve innate immune homeostasis and decrease the incidence of postoperative infectious complications.^5^ Undoubtedly IAP is only one of several determinants, as surgical tissue injury and anesthesia both constitute a hit to immune homeostasis. Innate immune cells possess a memory capacity referred to as trained immunity when it results in higher responsiveness and innate tolerance when cells become less responsive.^6^ The underlying metabolic and epigenetic reprogramming of immune cells occurs in reaction to stimulation. This concept has mainly been investigated and characterized in vaccinations, trauma, infectious diseases, sepsis, and atherosclerotic cardiovascular disease, but may also be relevant in the perioperative period. Further exploration of the innate immune phenotype, trained immunity, and innate tolerance in the perioperative period may identify patients more likely to develop susceptibility to infection. Perioperative epigenetic alterations could explain the prolonged postoperative immune effects and, if identified, can provide opportunity for early intervention to decrease postoperative infections. These epigenetic alterations consist of changes in chromatin structure and conformation, that regulate how easily genes are transcribed reviewed in.^7^ Potential immune influencing factors are challenging to separate or control for in perioperative clinical trials. Moreover, quantifying surgical tissue injury is challenging. There are numerous different damage-associated molecular patterns (DAMPs; endogenous proteins or fragments of cells that are released in circulation when a cell is damaged, or can be expressed on the threat of damage), and a few have been linked to immune dysregulation in surgical patients.^8^ Nonetheless, which DAMPs are most influential and the extent of their impact is still uncertain. Therefore, analysis of individual possible determinants of immune dysregulation in in vitro experiments can prove valuable.

**Figure 1.**
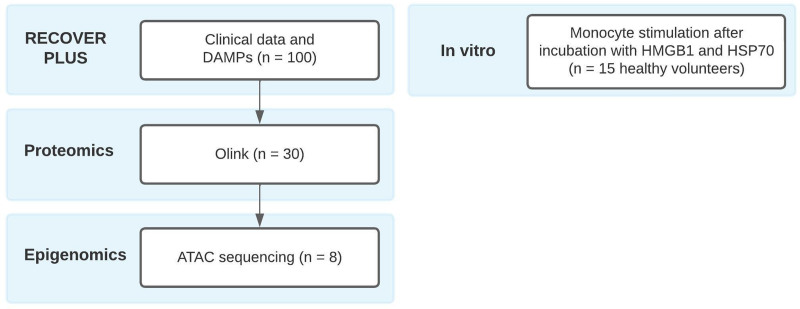
Overview of the RECOVER PLUS analyses and in vitro experiments. ATAC indicates assay for transposase-accessible chromatin; DAMPs, damage-associated molecular patterns; HMGB1, high-mobility group box 1; HSP70, heat shock protein 70; Olink, proximity extension immunoassay.

A better understanding of perioperative innate immunity is crucial to diminish the number of infectious complications after colorectal surgery. As monocytes are known to have an important role in the immune response associated with (surgical) trauma^9,10^ we aim to explore if epigenetic alterations in monocytes in response to surgery could explain increased postoperative susceptibility to infections. To this end, we will investigate (1) pre- to postoperative DAMP kinetics as well as the impact of DAMPs on innate immune dysregulation, and (2) the pre- to postoperative proteomic and epigenomic changes in the innate immune response. Given the smaller sample size for the subgroup analyses, these results should be interpreted as hypothesis-generating.

## METHODS

The RECOVER PLUS study (clinicaltrials.gov NCT03572413, principal investigator M. Warlé) is an immunological substudy of the multicenter double-blinded randomized RECOVER trial (clinicaltrials.gov NCT03608436), assessing the effects of low IAP facilitated by deep neuromuscular blockade on quality of recovery and innate immune homeostasis. Trials were registered before patient enrollment on June 18, 2018. The RECOVER trial included 178 patients in 3 different teaching hospitals, the first 100 patients enrolled at the Canisius Wilhelmina Hospital were included in the RECOVER PLUS substudy. Patients in the substudy were not treated differently, but blood was drawn before surgery and on postoperative day (POD) 1 and 3. The complete study protocol^11^ and primary analysis of the RECOVER trial^5^ were published previously. This manuscript adheres to the applicable Equator guidelines. An overview of the RECOVER PLUS analyses is shown in Figure 1, relevant methods are summarized below. The study was approved by the Medical Research Ethics Committee “CMO region Arnhem-Nijmegen” and the competent authority (Central Committee on Research Involving Human Subjects). All participants provided written informed consent for study participation. Additional in vitro experiments were performed in monocytes isolated from buffy coats from healthy volunteers obtained after written informed consent (Sanquin Blood Bank).

### Intervention and Patient Samples

Patients undergoing elective colorectal surgery were randomized in a 1:1 fashion to low IAP (8 mm Hg) and deep neuromuscular blockade (Post Tetanic Count [PTC] 1–2) or standard IAP (12 mm Hg) and moderate neuromuscular blockade (Train of Four [TOF] 1–2). Blood was drawn for all 100 substudy patients by venepuncture before surgery, at the end of surgery after neuromuscular block reversal, and on POD 1 and 3 if patients were still admitted at that time. Lithium heparin (LH) and ethylenediaminetetraacetic acid (EDTA) anticoagulated blood were centrifuged and plasma was stored at –80 °C until analysis.

### DAMPs, RNA Expression, and Ex Vivo Cytokine Production Capacity

Plasma DAMPs were measured from doubly centrifuged EDTA anticoagulated blood as previously described.^8,12^ In short, concentrations of heat shock protein 70 (HSP70; R&D Systems) and high-mobility group box 1 (HMGB1; IBL International GmbH) were measured batchwise by enzyme-linked immunosorbent assay (ELISA) according to manufacturer’s instructions. DNA was isolated with the QIAamp DNA Blood Midi Kit (Qiagen) and levels of nuclear deoxyribonucleic acid (nDNA) and mitochondrial DNA (mtDNA) were determined by quantitative polymerase chain reaction (qPCR) on a CFX96 real-time PCR Detection System using SYBR green reagents (Bio-Rad Laboratories) and expressed as fold-change relative to preoperative values of the same patient using the formula 2 Ct. Levels of nDNA were quantified using primers for glyceraldehyde 3-phosphate dehydrogenase (GAPDH): forward 5′-AGCACCCCTGGCCAAGGTCA-3′ and reverse 5-CGGCAGGGAGGAGCCAGTCT-3′. For the quantification of mtDNA levels, primers for MT-ND1 were used: forward 5′-GCCCCAACGTTGTAGGCCCC-3′ and reverse 5′AGCTAAGGTCGGGGCGGTGA-3′. RNA was isolated from blood collected in Paxgene RNA tubes using the Paxgene Blood RNA kit (Qiagen) and transcribed into cDNA using the iScript cDNA Synthesis kit (Bio-rad). Levels of RNA were quantified using 5′-AGGGCAGAATCATCACGAAGT-3′ forward primers and 5′-AGGGTCTCGATTGGATGGCA-3′ reverse primers for vascular endothelial growth factor A (VEGFA) and “5-AGTCCCTGTGCTAGGATTTTTCA-3” forward and “5-ACATAAACTCGCCTGATTGGTC-3” reverse primers for HLA-DRA. 18S was used as a reference gene with forward 5′-AAACGGCTACCACATCCAAG-3′ and reverse “5-CGCTCCCAAGATCCAACTAC-3” primers.

Whole-blood ex vivo cytokine production capacity on endotoxin stimulation was quantified as previously described.^10,11^ Briefly: 0.5 mL of LH anticoagulated whole-blood was added to preprepared tubes with 2 mL culture medium (negative control) and 2 mL culture medium supplemented with 12.5 ng/mL Escherichia coli lipopolysaccharide (LPS; serotype O55:B5 Sigma-Aldrich) resulting in a final concentration of 10 ng/mL. After mixing, tubes were cultured for 24 hours at 37°C, then centrifuged for 5 minutes at 1500 rpm and supernatants were stored at −80°C until analysis. Cytokines were measured in the supernatant by ELISA (R&D Systems) according to the manufacturer’s instructions.

### In Vitro Experiments

Peripheral blood mononuclear cells (PBMCs) from healthy volunteers were isolated by Ficoll-Paque (GE Healthcare) density gradient centrifugation and washed 3 times with phosphate-buffered saline (PBS) at 4°C. Monocytes were further purified from the PBMC fraction using the Pan-Monocyte Isolation Kit (MACS Miltenyi) according to the manufacturer’s instructions. All experiments were performed in Roswell Park Memorial Institute (RPMI) 1640 Dutch Modified (Gibco, Thermo Scientific) cell culture medium, supplemented with gentamycin 50 µg/mL, pyruvate 1 mM, and GlutaMAX 2 mM. Cells were cultured at 5 × 10^5^/well in a 96-well plate and incubated with culture medium only (negative control), HMGB1 (Merck), and HSP70 (Enzo Life Sciences) at various concentrations. Directly after, cells were stimulated with 10 ng/mL *Escherichia coli* LPS (serotype O55:B5; Sigma-Aldrich) for 24 hours. Supernatants were stored at −20 °C until analysis of interleukin 1β (IL-1β), IL-6, IL-10, and tumor necrosis factor (TNF) by ELISA (R&D Systems).

### Proteomics

Preoperative and POD1 plasma samples were used for commercial targeted plasma proteomics analysis (Olink). Olink Target 96 Inflammation panel was used to measure 92 inflammation protein biomarkers by multiplex proximity extension assays, as quantified by real-time PCR (qPCR).^13^ The thirty patients consisted of the 8 patients from epigenomic analysis and 22 patients randomly selected from all patients enrolled in the initial randomized controlled trial.

### Epigenomics

Genome-wide profiling of chromatin accessibility landscapes was performed on isolated monocytes using the Assay for Transposase-Accessible Chromatin using sequencing (ATAC-seq) as previously described.^14^ This technique can identify where the conformational changes in chromatin occur and whether it leaves the surrounding genes more (“open”) or less (“closed”) accessible for transcription. Comparing the chromatin structure from before and after surgery allows to determine which genes are influenced by surgery and/or anesthesia. PBMCs were isolated, monocytes were purified and ~50.000 were tagmented for each sample. Libraries were sequenced with Nextseq 500, FASTQ files were processed with the ENCODE pipeline on TERRA. Differentially accessible regions were identified by “edgeR,” peaks were annotated using “ChIPseeker” and gene ontology enrichments with “clusterProfiler.” Processed promoter capture Hi-C (PHi-C) data were downloaded from https://osf.io/u8tzp, coordinates were converted from hg19 to hg38 with liftOver tools. HOMER was utilized for motif enrichment. Differentially accessible regions were used as foreground and all detected peaks were used as background. The 8 patients were 4 consecutive patients from the low-pressure arm and 4 consecutive patients from the standard-pressure arm.

### Statistical Analyses

All statistical analyses were performed using Statistical Package for the Social Sciences (IBM SPSS Statistics, version 27). Pre- to postoperative levels of DAMPs (HMGB1 and HSP70) and in vitro cytokine production capacity after incubation with HMGB1 and HSP70 were compared with a Friedman test with post hoc Dunn’s multiple comparisons test. For analysis of ex vivo cytokine production capacity, a mixed-effects model analysis was used due to the missing values on POD3.

**Table 1.** Characteristics of All Substudy Patients and Patients Included in the Proteomic and Epigenomic Analyses

	All patients N = 100	Proteomic analysis N = 30	Epigenomic analysis N = 8
Gender (male/ female)	64/ 36	19/ 11	5/ 3
Age (y)	69 ± 9	69 ± 10	67 ± 9
BMI (kg/m^2^)	26.6 ± 4.4	25.5 ± 3.6	25.7 ± 2.1
ASAphysical status (I/ II/ III)	22/ 60/ 18	7/ 20/ 3	3/ 4/ 1
Intraabdominal pressure (8/12 mm Hg)	50/ 50	16/ 14	4/ 4
Type of surgery	39 30 18 8 3 2		
Sigmoid resection Right hemicolectomy LAR/ PME Left hemicolectomy Ileocecal resection Right hemicolectomy and sigmoid resection		10 8 7 3 2 0	2 5 1 0 0 0
Malignancy (yes/ no)	87/ 13	25/ 5	8/ 0

Presented values are absolute numbers or mean ± SD.

Abbreviations: ASA, American Society of Anesthesiologists; BMI, body mass index; LAR, low anterior resection; PME, partial mesorectal excision; SD, standard deviation.

**Table 2.** Infectious Complications

Infectious complications	Total	In proteomic analysis	In epigenomic analysis
Infected hematoma	3	1	1
Pneumonia	3	1	0
Abdominal infection/abscess	3	2	0
Anastomotic leak	2	1	0
Wound infection	2	2	0
Urinary tract infection	2	1	0
Total	15	8	1

**Figure 2.**
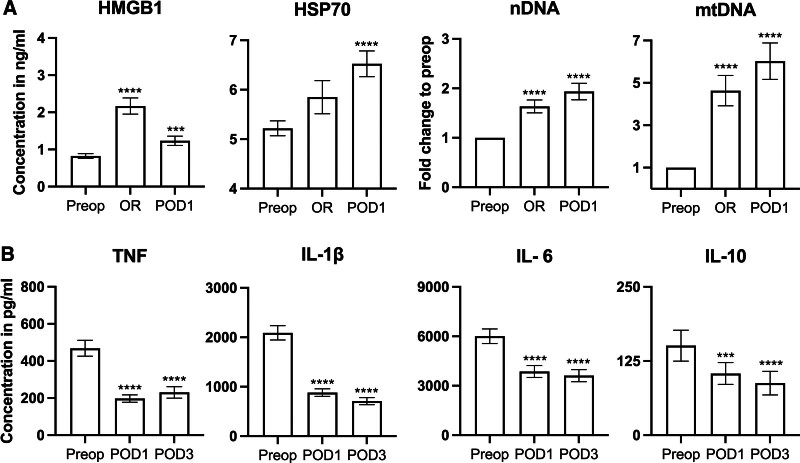
DAMPs and ex vivo cytokine production capacity. A, Changes in plasma HMGB1 and HSP70 from preoperative (preop) to the end of surgery (OR) and POD1 (comparisons were made with preoperative by Friedman test with post hoc Dunn’s multiple comparisons test), and fold-change in relation to preoperative values (Wilcoxon signed-rank test) for serum nDNA and mtDNA (n = 100). B, Ex vivo cytokine production capacity on stimulation of whole-blood with LPS preop (n = 100), POD1 (n = 99), and POD3 (n = 67; comparisons were made with preoperative by mixed-effects model analysis with post hoc Dunnett’s multiple comparisons test, *** *P* ≤.001, ****P ≤ .0001). Error bars display the SEM. HMGB1 indicates high-mobility group box 1; HSP70, heat shock protein 70; LPS, lipopolysaccharide; mtDNA, mitochondrial DNA; nDNA, nuclear DNA; OR, operating room; POD1, postoperative day 1; SEM, standard error of the mean.

**Figure 3.**
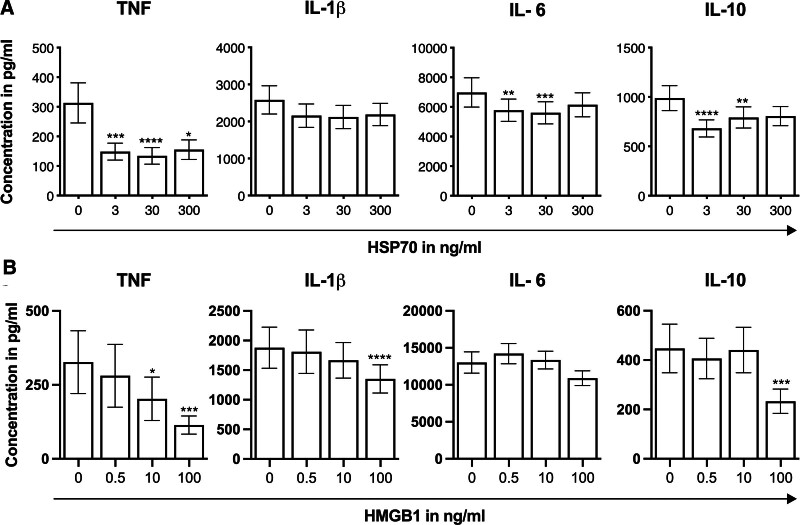
In vitro effects of HMGB1 and HSP70 on monocyte function. A, In vitro cytokine production capacity of healthy donor monocytes on LPS stimulation after preincubation with HSP70 (n = 15). B, HMGB1 (n = 15; comparisons were made with baseline by Friedman test with post hoc Dunn’s multiple comparisons test, *P < .05, **P ≤.01, ***P ≤.001, ****P ≤ .0001). Error bars display the SEM. HMGB1 indicates high-mobility group box 1; HSP70, heat shock protein 70; LPS, lipopolysaccharide; SEM, standard error of the mean.

**Figure 4.**
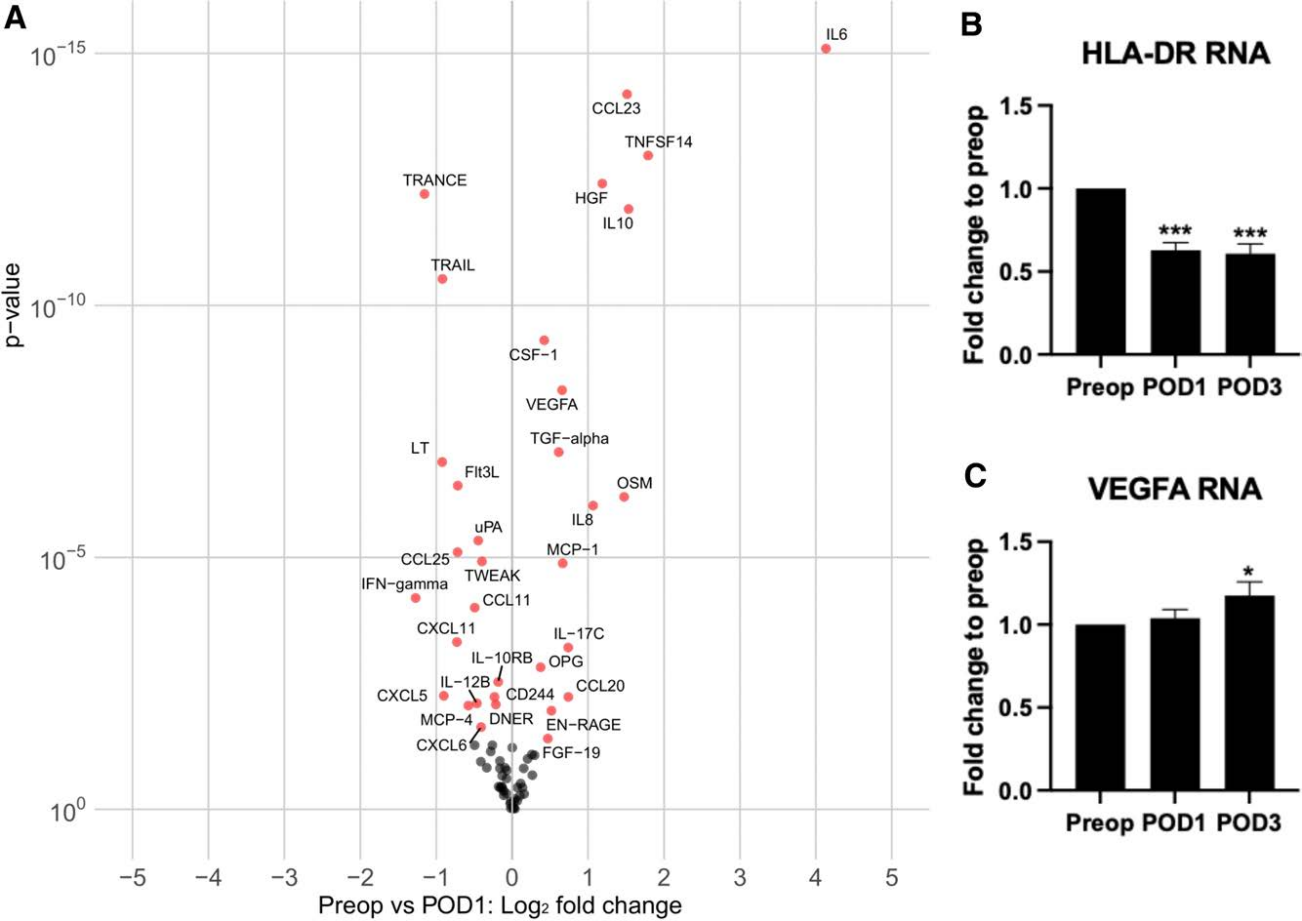
Pre- to postoperative proteomic changes. A, Volcano plot of the pre- to postoperative differences in proteins measured with the Olink inflammation panel, the *X*-axis denotes relative fold-change in expression compared to preoperative levels, the *Y*-axis represents the degree of statistical significance as tested with a paired Wilcoxon signed-rank test, corrected for multiple testing by the Benjamini-Hochberg procedure. The proteins marked in red are statistically significant at an FDR-adjusted *P*-value of < .05, the proteins marked in black are not. B, RNA levels of HLA-DR decrease from preoperative (preop) to POD1 and POD3. C, RNA levels of VEGFA are increased on POD3 compared to preoperative (preop; Wilcoxon signed-rank test; n =100, * = *P* < .05, ***P ≤.001). Error bars display the SEM. FDR indicates false discovery rate; HLA-DR, human leukocyte antigen – DR isotype; POD1, postoperative day 1; POD3, postoperative day 3; RNA, ribonucleic acid; SEM, standard error of the mean; VEGFA, vascular endothelial growth factor A.

qPCR fold-change values (nDNA, mtDNA, human leukocyte antigen – DR isotype [HLA-DR], and VEGF) were compared by a paired Wilcoxon signed-rank test. Pre- to postoperative differences of the Olink analysis were compared by a paired Wilcoxon signed-rank test and the false discovery rate (FDR) was adjusted using the Benjamini-Hochberg procedure. A value of *P* < .05 was considered significant and indicated in the graphs as *P ≤.01 as **P ≤.001 as *** and *P* ≤.0001 as ****.

## RESULTS

The screening and treatment allocation flowchart of the RECOVER study was included in the primary publication.^5^ For the substudy, 100 patients were analyzed irrespective of treatment allocation to assess pre- to postoperative differences. The patient characteristics of all patients, patients included in the proteomic and epigenomic analysis are displayed in Table 1, infectious complications are presented in Table 2.

### DAMPs and Ex Vivo Cytokine Production Capacity

Figure 2A shows a significant increase in circulating concentrations of HMGB1, HSP70, nDNA, and mtDNA at the end of surgery (operating room [OR]) compared to before surgery (preop). HMGB1 showed a mean 235% increase from preop to the end of surgery (95% confidence interval [CI] [166–305], *P <* .0001) and 90% increase (95% CI [63–118], *P =* .0004) preop to POD1. HSP70 increased by a mean 12% from preop to the end of surgery (95% CI [3–21], not significant [NS]) and 30% to POD1 (95% CI [18–41], *P <* .0001). nDNA increases by 66% (95% CI [40–92], *P <* .0001) at the end of surgery and 94% on POD1 (95% CI [60–127], *P <* .0001). mtDNA increases by 370% at the end of surgery (95% CI [225–515], *P <* .0001) and by 503% on POD1 (95% CI [332–673], *P <* .0001). Figure 2B illustrates a significant postoperative decrease in cytokine production capacity compared to before surgery for TNF, IL-1β, IL-6, and IL-10 on POD1 and POD3.

### In Vitro Effects of HMGB1 and HSP70 on Monocyte Function

To investigate the effects of circulating DAMPs on cytokine production, we preincubated monocytes of healthy volunteers (n = 15) with different concentrations of HSP70 and HMGB1 (Figure 3). In vitro incubation of monocytes with 30 ng/ml HSP70 decreased cytokine production capacity of TNF by 46% (95% CI [29–64], *P <* .0001), IL-6 by 22% (95% CI [12–32], *P =* .0004) and IL-10 by 19% (95% CI [12–26], *P =* .0015). In vitro incubation with 100 ng/ml HMGB1 decreased cytokine production capacity of TNF by 34% (95% CI [3–65], *P =* .0003), IL-1β by 24% (95% CI [16–32], *P <* .0001), and IL-10 by 40% (95% CI [21–58], *P =* .0009).

### Pre- to Postoperative Proteomic Changes

Using the Olink proteomics inflammation panel, 92 protein biomarkers were measured in the preoperative and POD1 plasma samples of 30 patients. The comparison of relative expression levels of inflammatory proteins before and after colorectal surgery are represented in the volcano plot of proteins as displayed in Figure 4A. The *X*-axis denotes relative fold-change in expression compared to preoperative levels, whereas the *Y*-axis represents the degree of statistical significance. Proteins with a significantly changed expression from pre- to postoperative are marked by name and in red. Figure 4A illustrates the elaborate surgical signature of simultaneous up-and downregulation of inflammatory proteins. Next, RNA expression of antigen-presenting MHC class II receptor HLA-DR and VEGFA were determined in blood from 100 patients obtained preoperatively, as well as on POD1 and POD3. RNA expression of HLA-DR was significantly decreased on POD1 and POD3 (Figure 4B). RNA expression of VEGFA was only elevated on POD3 (Figure 4C).

### Pre- to Postoperative Epigenomic Changes

**Figure 5.**
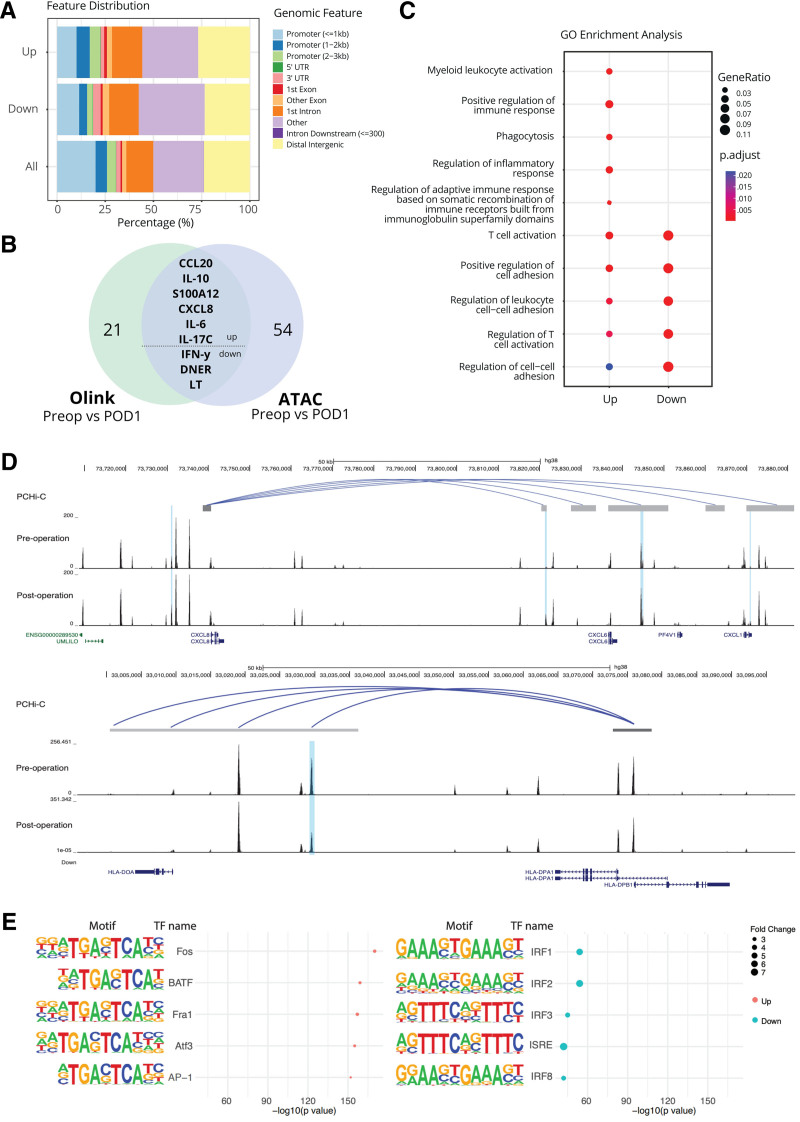
ATAC-seq analysis reveals differential accessibilities and key transcription factors. A, Genomic distribution of differential accessible regions in loci exhibiting increased (up) or decreased (down) accessibility. B, Of the 21 significantly changed proteins identified with Olink and 54 significantly altered genes identified with ATAC seq, 9 overlapped. C, GO enrichment analysis of differentially accessible genes after surgery. The size of the dots indicates gene ratio and the color indicates adjusted *P*-value. D, Genomic view around CXCL8 (IL-8) and HLA-DPA1, the blue shaded regions indicate differential accessibility (more accessible for CXCL8 and less accessible for HLA-DPA1 The PCHi-C track indicates interactions between the promoter of CXCL8 and HLA-DPA1 and other elements. Dark gray bars are bait regions and light gray bars are open end regions. The interactions with CHICAGO score >5 in monocytes are only shown. Accessibilities of pre- and postoperation in monocytes from one of the patients are shown below PCHi-C track. E, Top 5 enriched motifs in differentially accessible regions. *X*-axis indicate –log10 transformed *P*-value. The size of dots means fold enrichment. ATAC-seq indicates Assay for Transposase-Accessible Chromatin; CHICAGO, Capture Hi-C analysis of genomic organisation; CXCL8, C-X-C motif ligand 8; GO, gene ontology; HLA-DPA1, human leukocyte antigen - DPA1; IL-8, interleukin 8; Olink, proximity extension immunoassay; PCHi-C, promoter capture Hi-C.

To elucidate the mechanisms governing the observed alterations in gene expression, we performed the ATAC sequencing on isolated monocytes from 8 patients, both preoperatively and on POD1. By assessing the differences in chromatin structure, we can assess genomic accessibility, as the conformational changes leave certain DNA regions more or less accessible for transcription. By investigating whether this occurs for inflammatory genes, we can deduct whether these changes underlie postoperative immune suppression. This analysis revealed 2793 loci exhibiting increased accessibility after surgery, while 610 regions demonstrated decreased accessibility. Differentially accessible loci were predominantly located in intergenic regions as compared to other genomic regions (Figure 5A), potentially suggesting that changes in enhancer accessibility are instrumental in driving postoperative gene expression alterations. In alignment with the Olink data, our findings indicate significant changes in accessibility of inflammatory genes, including CXCL8 (IL-8; Figure 5B; shows which genes are up-and downregulated, the same direction in Olink and ATAC analysis, Figure 5C, 5D). Conversely, we observed reduced accessibility of antigen presentation genes in postoperative samples (Figure 5B, 5D).

Including patients from both the low and standard-pressure arm may have introduced heterogeneity to these results. Although the number of investigated patients was small, we explored whether the intervention was influenced by principal component analysis (Supplemental Digital Content 1, Supplemental Figure 1, http://links.lww.com/AA/F90). Only 56 loci (out of 157,471 consensus peaks in autosome; FDR <0.01) were differentially accessible when comparing low versus standard pressure. Whereas, 3602 loci (out of the consensus peaks) were differentially accessible from pre- to postsurgery. Low and standard pressure conditions did not lead to easily distinguishable changes in the epigenomic landscape as evidenced by the close proximity and overlap of the data points representing the 2 conditions.

To identify whether enhancers play a role in modulating the identified genes to have an altered accessibility after surgery, we utilized publicly available PHi-C data to determine whether differentially accessible enhancers are predictive of contacting the promoter of 1 gene with enhanced accessibility (CXCL8) and 1 with reduced accessibility (HLA-DPA1; Figure 5D). The 4 out of the 5 enhancers predicted to interact with the CXCL8 promoter were also shown to have an increase in differential accessibility (blue bars). Meanwhile for HLA-DPA1, 1 out of the 4 predicted enhancers showed a change in accessibility.

Transcription factors (TFs) are proteins that modulate gene expression and chromatin accessibility through direct DNA binding. Given that TFs recognize specific DNA sequences, we assessed whether recognition sequences for any particular TF were enriched within differentially accessible loci (Figure 5E). Our analysis revealed a significant enrichment of AP-1 family motifs in loci exhibiting increased accessibility in postoperative samples. Conversely, we identified a high enrichment of interferon regulatory factor (IRF) family protein motifs in loci exhibiting decreased accessibility.

## DISCUSSION

In this substudy, we show the major pre- to postoperative changes in DAMPs and inflammatory mediators after laparoscopic colorectal surgery. For several of these circulating inflammatory markers, we have identified concomitant epigenetic alterations in monocytes. This supports our hypothesis that surgical tissue injury and/or anesthesia induce epigenetic and functional changes (immune tolerance) in monocytes, which play a substantial role in postoperative immune dysregulation. If these alterations last for days to several months, as described for other stimuli,^15^ these monocyte pathways could provide leads for treatment targets to prevent infectious complications after colorectal surgery.

It is well known that surgery with general anesthesia leads to immune dysregulation. Nevertheless, it remains difficult to determine the cause: is it mainly tissue injury, the anesthetics and analgesics, perioperative stress and pain, or a combination of all? The theory that DAMPs as ligands of toll-like receptors (TLRs) on immune cells acts as danger signals and modify the innate immune response was already presented almost 3 decades ago by Matzinger.^16^ This association has been confirmed in patient studies^8,17^ and in vitro experiments.^18,19^ It is well established that DAMPs can induce trained immunity through epigenetic regulation of transcriptional programs.^20^

Here, we show a reduction in cytokine production capacity of human monocytes for TNF, IL-6, and IL-10 on incubation with HSP70, and of TNF, IL-1β, and IL-10 after incubation with HMGB1. In postoperative patients, we found mean circulating HSP70 levels above 6 ng/ml, whereas in vitro the significant suppressive effects on TNF, IL-1β, IL-6, and IL-10 production capacity are already seen at 3 ng/mL. Several of the measured changes are not large. Nonetheless, they are comparable with changes in DAMPs and cytokines reported after HIPEC surgery, where such changes were associated with immune suppression and infectious complications.^8^ The physiological relevance of these changes cannot be definitively established. However, it is important to consider the cumulative and synergistic effects of multiple DAMPs and cytokines in vivo. Each measured DAMP and cytokine is one of many, and although only a small selection is presented here, there are hundreds of different DAMPs that bind to pattern recognition receptors like TLRs. A collective increase in these DAMPs can lead to significant downstream effects.

The significantly altered proteins overlapping from proteomic and epigenomic analyses are IL-6, IL-10, CXCL8 (IL-8) and IL-17C, CCL20, S100A12 (EN-RAGE), interferon-gamma (IFN-γ), and lymphotoxin (LT). Multiple studies highlight the association between higher postoperative increases of interleukins IL-6, IL-8, and IL-10 concentrations and increased risk of sepsis.^21,22^ A systematic review of IL-6 in over 5000 surgical patients with cancer of the gastrointestinal tract reveals high IL-6 is associated with impaired overall survival.^23^ IL-17C is involved in host defense and mainly produced by epithelial cells on bacterial challenge or inflammatory stimuli, IL-17C deficient mice are highly resistant to endotoxin (LPS) induced septic shock.^24^ Chemokine C-C motif ligand 20 (CCL20) is a ligand for C-C chemokine Receptor 6 (CCL6) expressed in colon, liver, and skin. Binding and activation of the CCL20-CCL6 axis is not only associated with inflammation and infections but also with cancer proliferation by modulation of the tumor microenvironment through immune cell control.^25^ Alarmin S100A12 positively correlates with length of intensive care unit (ICU) stay, 28-day mortality and in-hospital mortality after major abdominal surgery.^26^ IFN-y and LT concentrations are downregulated while they seem to exert protective properties against postoperative immune suppression.^27^ Patients with LT polymorphisms undergoing major gastrointestinal surgery have a higher risk of postoperative complications and mortality for patients with postoperative sepsis.^28^ For all of these factors, more profound dysregulation is associated with a worse patient outcome. Accordingly, potential interventions should be aimed at reducing postoperative immune dysfunction.

In critical illness, innate immune dysfunction is often quantified by 2 parameters: LPS-induced whole-blood *ex vivo* cytokine production capacity and monocyte MHC class II (like HLA-DR) expression.^29^ The identified enrichment of IRF family protein motifs in loci exhibiting decreased accessibility and activating protein-1 (AP-1) family motifs in loci exhibiting increased accessibility can contribute to postoperative impairment in both parameters. IRF’s regulate TLR and IFN signaling, and thereby have a crucial role in the response to pathogen infection, inflammation, antigen-presentation, antimicrobial defense, and tumor suppression (reviewed in^30^). IRF’s recognize the interferon-stimulated response element (ISRE, which also exhibits decreased accessibility) in promoters of target genes.^31^ Binding of pathogen- or damage-associated molecular patterns (PAMPs or DAMPs) to TLRs or IFN to the IFN receptors induces IRF activation and translocation to the nucleus where they interact with coacting TFs like STAT, NF-kB and PU.^28^ Overexpression of IRF’s is implicated in auto-immune disease,^32^ the postoperative decreased accessibility of IRF’s observed in this study likely contributes to immunosuppression. Interferon signaling activates IRF family proteins, and expression and activation of IRF1, 2, and 8 is dependent on IFN-y.^33^ Proteomic and epigenomic analysis revealed a postoperative decrease in IFN-γ expression. The top 5 enriched motifs in loci exhibiting increased accessibility (Fos, BATF, FRA1, Atf3, and AP-1) are all subunits or dimers of the AP-1 protein family.^34^ AP-1 activity is regulated by physiological and pathological stimuli like cytokines, stress signals, infections, and oncogenic stimuli. AP-1 TFs are regulators of the immune response, they cooperate with TF nuclear factor of activated T-cells (NFAT) to regulate cytokine production and T-cell activation, and play a vital role in inflammatory disorders.^35^ Hyperinflammation and immune suppression often coincide during immune dysregulation, and likely exacerbate each other.^36^ Both IRFs and AP-1 are referred to as a double-edged sword with respect to the immune response,^34,37^ as both a shortage and surplus can negatively impact outcomes. This emphasizes the importance of only reducing excessive dysregulation without tipping the balance to the other side.

A limitation of this study is that the exploratory proteomic and epigenomic analyses were only performed in a small subgroup, as they are time-consuming and costly. Therefore, the results need to be interpreted as hypothesis-generating. Nevertheless, the trial findings are theoretically founded and confirmed by independent in vitro experiments which substantiate the conclusions. The subset of patients analyzed with Olink technology contained patients with and without postoperative infections, however, the number of patients was too small for reliable comparisons. RNA levels of HLA-DR showed a significant reduction, indicative of immune suppression and aligning with our clinical expectations. However, it is important to remember RNA levels do not always correlate with protein levels. Flow cytometry of HLA-DR could have provided superior insights by directly quantifying the protein expression levels, offering a more precise assessment of immune competence.In conclusion, our study endorses the relevance of epigenomic alterations in monocytes to immune dysregulation after laparoscopic colorectal surgery. Next, it is paramount to investigate how long postoperative innate immune tolerance lasts and to explore whether we can meaningfully modulate dysregulation through the identified pathways. Conceivably, not all patients will benefit from the same therapy as individual patient’s immune response and training or tolerance develop based on previous encounters with stimuli and pathogens. Moreover, immune training and tolerance seem to occur simultaneously in different pathways. First, it could prove valuable to investigate the effects of anesthetics on IRF and AP-1 accessibility. This is still relatively unexplored; sevoflurane seems to affect AP-1 expression in vitro in human kidney cells,^38^ while propofol upregulates IFN-y production in cultured NK cells.^39^ The knowledge on blocking or administering immune proteins has greatly advanced in recent years and is already being applied in several infectious diseases, like IL-6 blockade and IFN-y administration in coronavirus disease-2019 (COVID-19).^40,41^ Moreover, AP-1 inhibitors are increasingly studied as a potential cancer treatment.^34,42^ It is key to investigate how we can identify which patients could benefit from what treatment. Ideally in the future, we will be able to identify patients at higher risk for postoperative infectious complications and create a personalized perioperative plan to minimize postoperative immune dysregulation.

## DISCLOSURES

**Conflicts of Interest:** M. Warlé received grants from Merck Sharp & Dohme for investigator-initiated studies. No other authors declared Conflicts of Interest. **Funding:** RECOVER and RECOVER PLUS were supported by 2 research grants from the Investigator-Initiated studies Program from Merck Sharpe & Dohme, reference numbers #55890 and #57675. Sugammadex was provided for all study patients by Merck Sharpe & Dohme. **This manuscript was handled by:** Christina M. Pabelick, MD.

## Supplementary Material


