# Supplementary material for: Postoperative Innate Immune Dysregulation, Proteomic, and Monocyte Epigenomic Changes After Colorectal Surgery: A Substudy of a Randomized Controlled Trial
Source: Anesth Analg. 2024 Oct 25;140(1):185–96. doi: 10.1213/ANE.0000000000007297 (PMC11620323; doi:10.1213/ANE.0000000000007297)
Supplement: Supplementary file 1 [file ane-140-185-s001.docx]

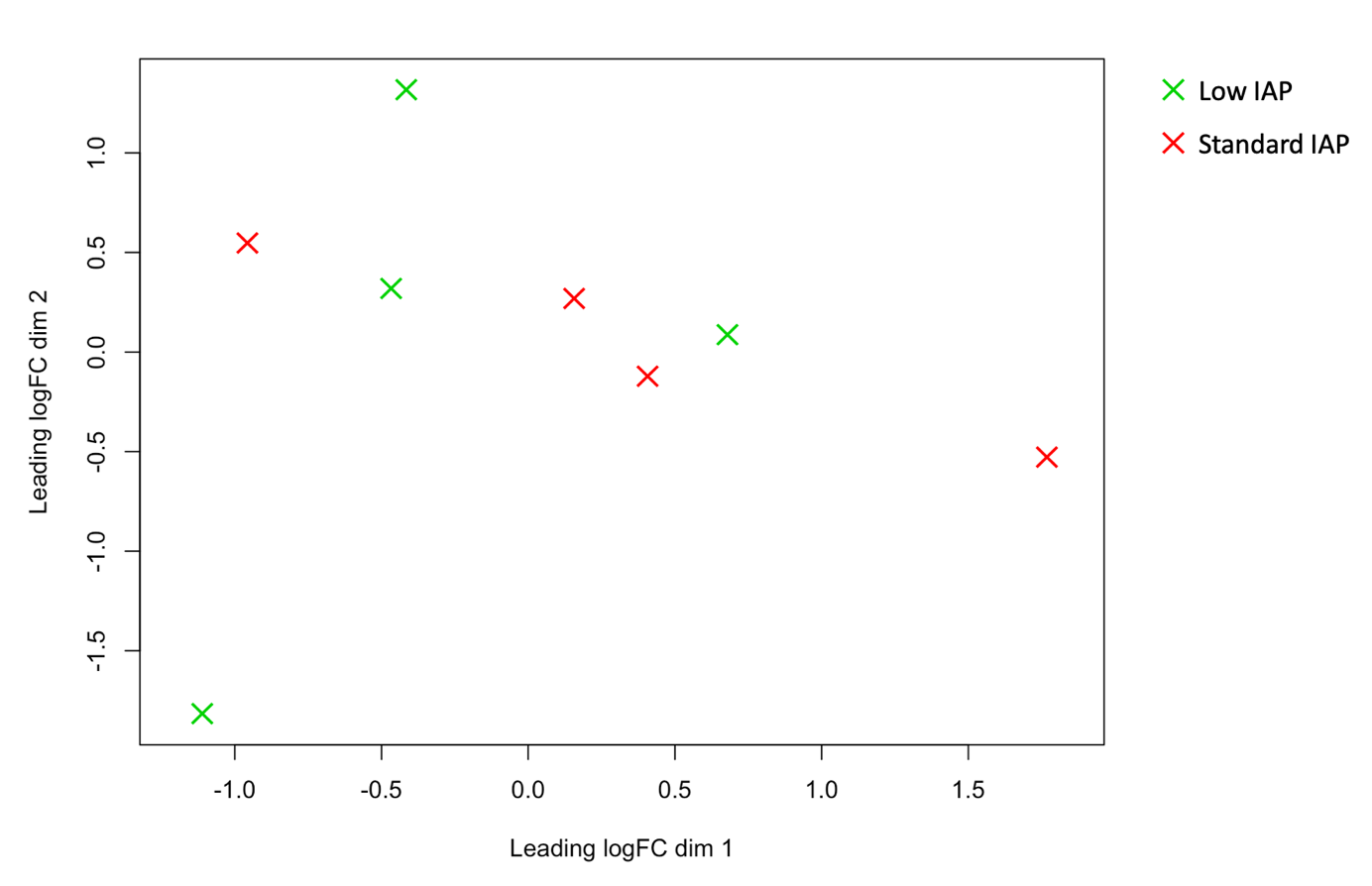


Supplemental Figure 1. Principal component analysis of low and standard pressure. Low and standard pressure conditions did not lead to easily distinguishable changes in the epigenomic landscape as evidenced by the close proximity and overlap of the data points representing the two conditions. Only 56 loci (out of 157,471 consensus peaks in autosome; False Discovery Rate < 0.01) were differentially accessible when comparing low versus standard pressure. Whereas, 3,602 loci (out of the consensus peaks) were differentially accessible from pre- to post-surgery.
